# Correction: Liponitroxides: EPR study and their efficacy as antioxidants in lipid membranes

**DOI:** 10.1039/c9ra90073j

**Published:** 2019-10-14

**Authors:** Giovanna Mobbili, Emanuela Crucianelli, Antonio Barbon, Massimo Marcaccio, Michela Pisani, Annalisa Dalzini, Eleonora Ussano, Marco Bortolus, Pierluigi Stipa, Paola Astolfi

**Affiliations:** Department of Life and Environmental Sciences, Università Politecnica delle Marche Via Brecce Bianche 12 I-60131 Ancona Italy; Department of Materials, Environmental Sciences and Urban Planning, Università Politecnica delle Marche Via Brecce Bianche 12 I-60131 Ancona Italy p.stipa@univpm.it; Department of Chemical Sciences, Università di Padova Via Marzolo 1 I-35131 Padova Italy marco.bortolus@unipd.it; Department of Chemistry “G. Ciamician”, Università di Bologna Via Selmi 2 I-40126 Bologna Italy; Department of Material Sciences, Università degli Studi di Milano Bicocca Via Roberto Cozzi 55 I-20125 Milano Italy

## Abstract

Correction for ‘Liponitroxides: EPR study and their efficacy as antioxidants in lipid membranes’ by Giovanna Mobbili *et al.*, *RSC Adv.*, 2015, **5**, 98955–98966.

The authors regret that an incorrect equation was shown in the original article. On page 98963 of the original article, in the equation for fitting power saturation data the term *x*^1/2^ was incorrectly shown as *p*_1/2_. The correct equation is shown below:*y* = *Ax*^1/2^[1 + (2^1/*h*^ − 1)*x*/*p*_1/2_]^−*h*^

The Royal Society of Chemistry apologises for these errors and any consequent inconvenience to authors and readers.

## Supplementary Material

